# Case Report: A Novel Lateral Approach to the C7, C8, and T1 Intervertebral Foramina for Resection of Malignant Peripheral Nerve Sheath Neoplasia, Followed by Adjunctive Radiotherapy, in Three Dogs

**DOI:** 10.3389/fvets.2022.869082

**Published:** 2022-06-03

**Authors:** Oliver Marsh, Naomi Shimizu, Sarah L. Mason, Ane Uriarte

**Affiliations:** ^1^Linnaeus Veterinary Limited, Neurology and Neurosurgery Service, Southfields Veterinary Specialists, Essex, United Kingdom; ^2^Linnaeus Veterinary Limited, Orthopaedic and Soft Tissue Surgery Service, Southfields Veterinary Specialists, Essex, United Kingdom; ^3^Linnaeus Veterinary Limited, Oncology Service, Southfields Veterinary Specialists, Essex, United Kingdom

**Keywords:** peripheral nerve sheath tumor, surgery, radiotherapy, dog, brachial plexus

## Abstract

This case report describes the diagnosis, management and outcome of three dogs with peripheral nerve sheath tumors (PNSTs) involving the brachial plexus, C7 (case 1), C8 (case 2), and C8 and T1 (case 3) spinal nerves and nerve roots with intrathoracic invasion. Surgical resection required thoracic limb amputation and removal of the first rib, facilitating a novel lateral approach to the spinal nerves and foramina in all cases. This was followed by hemilaminectomy and rhizotomy in cases 1 and 2. Adjunctive radiotherapy was then performed in all dogs. All three dogs regained a good quality of life in the short-term following surgery. Two were euthanased after 3 and 10 months, following detection of a pulmonary mass in one case and multiple thoracic and abdominal masses in the other. The third dog was alive and well at the time of writing (7 months post-surgery). This surgical approach facilitated good access and allowed gross neoplastic tissue to be resected. The ease of surgical access was dependent, to a degree, on the size of the patient. This surgical approach can be considered in cases of PNSTs involving the caudal cervical or cranial thoracic spinal nerves and nerve roots. Adjunctive radiotherapy should be considered as part of a multi-modal approach to these challenging tumors due to the difficulty of achieving clean margins, particularly proximally, even with optimal surgical access.

## Introduction

Peripheral nerve sheath tumors (PNSTs) are malignant mesenchymal tumors arising from the myelin sheath or connective tissues surrounding nerves. Historically, terms such as Schwannoma and neurofibroma have been used (1, 2). Since 2013, World Health Organization guidelines have suggested the use of the term peripheral nerve sheath tumor in human medicine (3), and veterinary nomenclature has followed this lead.

In dogs, PNSTs most commonly affect the brachial plexus and its contributing spinal nerves, and less frequently the pelvic limb (4). Typical clinical signs are insidious and include progressive lameness, monoparesis, muscle atrophy, a palpable mass and pain of variable severity (4–9). A presumptive diagnosis is usually achieved *via* CT (7) or MRI (8), or less commonly ultrasound (10), with or without confirmation *via* biopsy or fine needle aspirate (11).

Common treatment options for PNSTs include surgery with or without radiotherapy, and palliative therapy. Optimal treatment for canine brachial plexus tumors is not supported by strong evidence, and is also debated in human medicine (12). Surgery as the sole treatment in dogs has been associated with a poor prognosis due to short times to recurrence and death, reported to be 7.5 and 12 months, respectively (4). With nerve root involvement, the mean disease-free interval is 1 month and survival time around 5 months (4). However, short disease-free and survival times are not universal, and cases have been reported in which surgery has conferred prolonged survival and excellent quality of life. This appears to be particularly associated with complete mass resection (6). Where complete resection is not possible, there is some evidence that the use of adjunctive radiotherapy prolongs disease-free and survival times (13). Radiotherapy as a sole treatment appears to give a similar outcome to surgery alone (9).

Surgical resection is challenging when the caudal cervical and cranial thoracic spinal nerves and nerve roots are involved due to three main factors: the presence of the scapula, vertebral canal involvement and potentially intra-thoracic extension. A lateral approach to resect PNSTs involving the C6–C7, C7–T1, and T1–T2 intervertebral foramina, with intra-thoracic extension, has not been described. Here, we provide a description of the clinical presentation, imaging findings, surgical procedures, radiotherapy and outcome in three dogs with PNSTs involving the brachial plexuses, first rib and C7, C8, and T1 spinal nerves.

## Case 1

### Signalment and Clinical Presentation

A 12-year old, female neutered West Highland White Terrier weighing 9.25 kg presented with a 5-month history of progressive left thoracic limb lameness and pain that had shown no response to meloxicam and a mild, short-lived response to gabapentin. Neurological examination was limited due to the severity of the dog's pain. Left thoracic limb non-weight bearing lameness was observed. There was marked discomfort upon any attempt to touch the left thoracic limb. There was severe wastage of the left thoracic limb musculature. No other neurological deficits were present. Hematology and biochemistry profiles were unremarkable.

### Imaging

**Figure 1:**
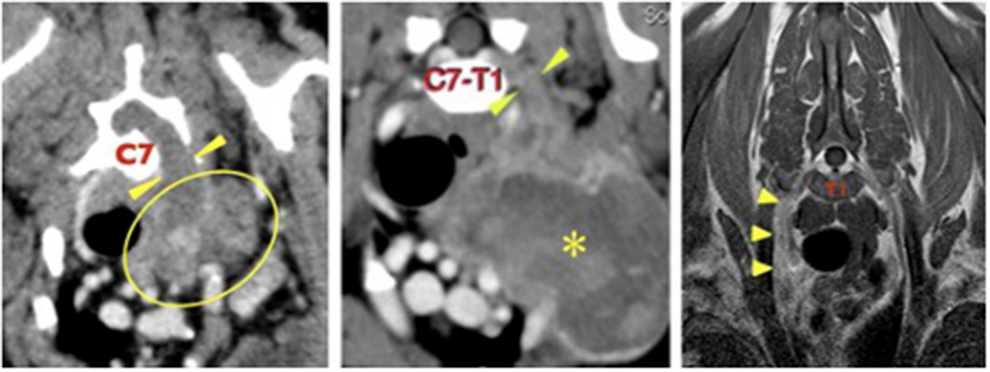
Left, case 1: transverse CT image at the level of the C7 vertebral body. There is a large, irregular soft tissue attenuating mass in the left axilla (outlined) which extends dorsally (arrowheads) through the enlarged C6–C7 intervertebral foramen to enter the vertebral canal. Middle, case 2: transverse CT image at the level of the C7–T1 intervertebral formamina showing a large, irregular mass (asterisk in its center) in the left axilla, extending proximally (arrowheads) through the C7–T1 intervertebral foramina to enter the vertebral canal. Right, case 3: transverse T1 weighted post-contrast image at the level of the mid T1 vertebral body. The right C8 spinal nerve (arrowheads) is enlarged and irregular and shows mild, homogenous contrast uptake. The right side of the dog is on the left side of each image.

A pre-referral CT scan of the thorax and thoracic limbs revealed a poorly defined, soft tissue attenuating mass extending from the left axilla dorsally as a thick cord toward the vertebral canal (Figure 1). It passed through the widened C6–C7 intervertebral foramen to form a mass that compressed and displaced the spinal cord to the right. The mass was closely associated with the first rib and invaded the entrance to the thorax. There was no evidence of pulmonary metastatic disease.

### Surgical Procedure

**Figure 2:**
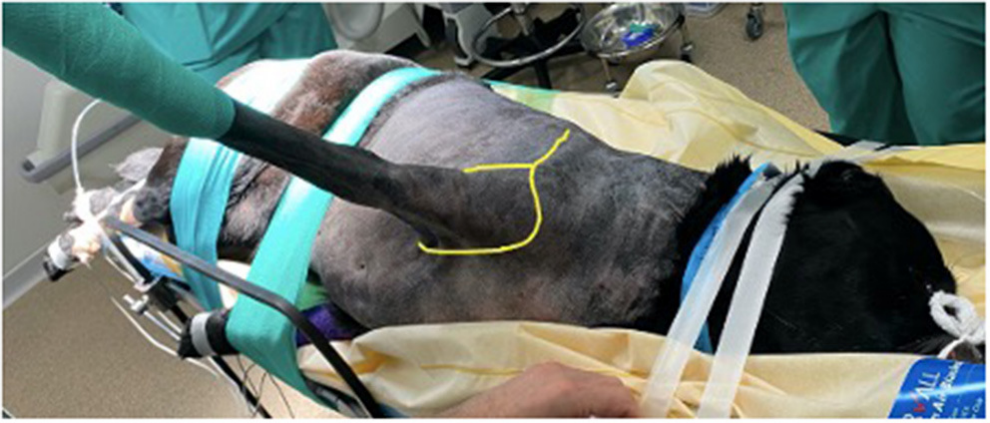
Patient positioning prior to surgery. Both thoracic limbs are retracted caudally to allow the surgeon closer and more comfortable access. The dog is held securely to the table using tape and elasticated bandage, to prevent movement when the table is tilted. Sandbags (not visible in this image) are placed under the dog's cervical region to maintain the vertebral column in horizontal alignment. The yellow line shows how the initial incision is performed- dorsoventrally over the spine of the scapula and then in a circumflex manner around the limb.

**Figure 3:**
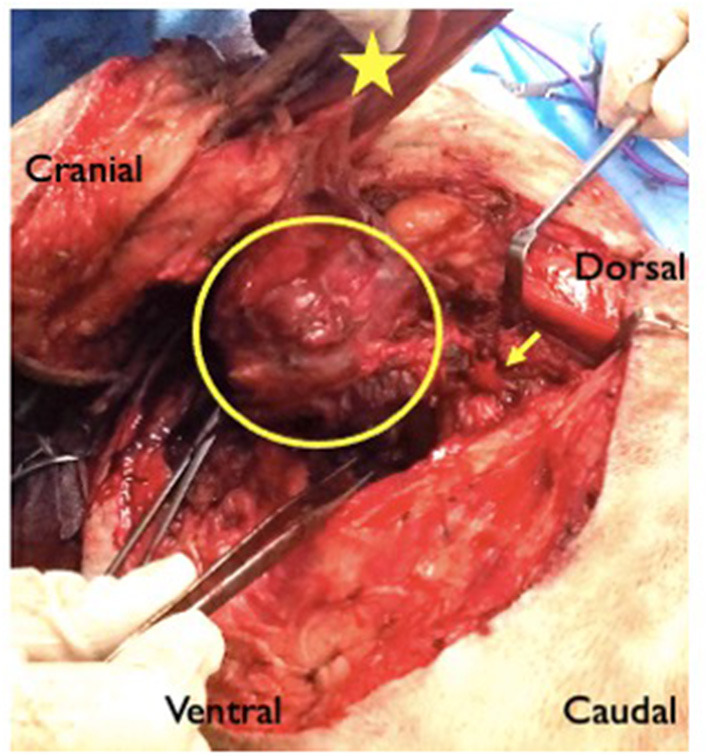
Intraoperative image from case 2 showing a large ovoid axillary mass (circled) with extension (arrow) toward the C7–T1 intervertebral foramen. The star shows the medial aspect of the scapula. The forceps in the surgeon's right hand show the region of the osteotomised rib.

Thoracic limb amputation and rib resection were performed following previously described techniques (14). An incision was made over the scapula and in a circumflex manner around the left thoracic limb (Figure 2). The cephalic vein was ligated and divided where it passed deep to the cleidobrachialis muscle. The omotransversarius, trapezius, rhomboideus and serratus dorsalis muscles were incised at their insertions on the scapula. At the caudal aspect of the axillary space, the insertions of the latissimus dorsi, teres major, and cutaneous trunci muscles were transected at their insertions on the teres tubercle of the humerus. With the dorsal aspect of the scapula held in abduction, the thoracodorsal, axillary and lateral thoracic arteries, and brachial, thoracodorsal, and axillary veins were ligated and transected. The superficial and deep pectoral muscles were transected from their insertions on the humerus. With the scapula abducted, the mass could be visualized medial to it. The carotid, jugular, esophagus and vagosympathetic trunk were identified and were not invaded by the mass. The mass was encircling the first rib. The rib and mass were isolated from surrounding soft tissue and the intercostal artery ligated dorsally and ventrally. The tubercule and head of the rib were disarticulated and an osteotomy was performed distally using bone cutters. A combination of sharp and blunt dissection was performed around the mass (Figure 3). The abnormal spinal nerve was identified and transected just ventral to the vertebral body allowing removal of the distal part of the mass, the rib and the limb. The table was tilted from horizontal to around 20 degrees toward the vertical plane, improving access and visualization for the following part of the surgery. The approach to the hemilaminectomy was made by bluntly dissecting, cutting or cauterizing the serratus ventralis, deep scalenus, longissimus cervicis, intertransversarii dorsalis, and intermedius cervicis muscles. A C6–C7 hemilaminectomy was then performed. The mass could be visualized compressing and invading into the spinal cord. A rhizotomy was performed as close to the spinal cord as possible and the abnormal spinal nerve was resected. The surgical site was copiously lavaged prior to placement of a chest drain and routine closure.

### Recovery and Follow-Up

The dog was bright and comfortable the day following surgery, was eating well and was able to stand unaided. The dog was discharged 2 days after surgery. Two weeks later, definitive intent radiotherapy was performed (51 Gy over 18 daily fractions). The dog was reported to be bright and happy at home for a further 6 weeks, before acutely becoming painful. There were no obvious neurological deficits on re-examination, but the dog was painful on being handled. Thoracic radiographs were performed which revealed a single 2.5 cm diameter well-defined soft tissue opacity in the right middle lung lobe most consistent with a neoplastic mass. The owner declined further investigations including advanced imaging and biopsy of the mass and instead requested euthanasia, declining post-mortem.

## Case 2

### Signalment and Clinical Presentation

A 7-year old, male entire, English Springer Spaniel weighing 20 kg presented with a 10 month history of progressive left thoracic limb lameness unresponsive to meloxicam and gabapentin. Examination revealed non-weight bearing lameness of the left thoracic limb. In this limb, the postural reactions were absent, the withdrawal reflex was incomplete and the muscle mass was markedly reduced. The cutaneous trunci reflex was present on the right and absent on the left. Miosis and third eyelid protrusion was seen in the left eye (partial Horner syndrome). A firm, round, painful mass was palpable in the left axilla. Hematology and biochemistry profiles were unremarkable.

### Imaging

Pre-referral CT of the neck, thorax, and elbows had revealed the presence of an ovoid, 8 × 7 cm diameter soft tissue attenuating mass in the left axilla, extending to the thoracic inlet where it displaced the trachea, esophagus, common carotid, and left subclavian arteries. It surrounded the left axillary artery and circumference of the first rib, where it was causing cortical lysis and periosteal reaction. The C8 spinal nerve was thickened and was extending into the vertebral canal where it was indenting the spinal cord (Figure 1). The axillary portion of the mass showed marked contrast enhancement and the abnormal C8 nerve showed mild contrast enhancement. There was no evidence of pulmonary metastatic disease.

### Surgery

Forequarter amputation and first rib removal were performed in the same manner as in case 1. The mass was followed proximally and the abnormal C8 spinal nerve identified, prior to transecting it and removing the distal part of the mass and the limb. The approach to the hemilaminectomy site was as in case 1. A left C7–T1 hemilaminectomy was then performed. The enlarged C8 nerve roots were resected ~1–2 mm distal to their attachment to the spinal cord, which appeared macroscopically normal. Lavage, chest drain placement and closure were performed as in case 1.

### Recovery and Follow-Up

The dog was comfortable the day following surgery and was discharged after 5 days. At 2-week re-examination there were no neurological abnormalities present and the Horner syndrome had resolved. Definitive intent radiotherapy was then performed (49.4 Gy over 19 daily fractions). Adjunctive metronomic chemotherapy (cyclophosphamide 50 mg/m^2^/48 h) and meloxicam (0.1 mg/kg/24 h) was started 3 months post-surgery. The dog was reported to be well, without obvious abnormalities, for 9 months, before becoming acutely dyspnoeic. Investigations revealed multiple thoracic and abdominal masses, which were not sampled, leading to euthanasia.

## Case 3

### Signalment and Clinical Presentation

A 10-year old, male neutered, Labrador weighing 39.7 kg was presented with a 3 month history of progressive right thoracic limb lameness and paresis. Examination revealed intermittent non-weight bearing lameness, severe paresis, spontaneous knuckling, absent postural reactions, reduced withdrawal reflex, and poor muscle mass in the right thoracic limb. No obvious discomfort or masses were detected on palpation. Hematology and biochemistry profiles were unremarkable. The clinical signs progressed despite 2-week courses of gabapentin (8 mg/kg/8 h) and prednisolone (0.5 mg/kg/24 h).

### Imaging

Pre-referral radiographs of the thorax, cervical spine, and thoracic limbs were normal. MRI of the cervical and cranial thoracic vertebral column, spinal cord and brachial plexuses revealed that the right C8 and T1 nerve roots, spinal nerves, and brachial plexus were enlarged and irregularly marginated, were STIR hyperintense, mildly T2 hyperintense and T1 isointense compared to normal muscle, and showed mild contrast enhancement (Figure 1). The abnormal T1 spinal nerve was closely associated with the first rib. No obvious spinal cord involvement was observed. There was no evidence of pulmonary metastatic disease.

### Surgery

Forequarter amputation and first rib removal were performed in the same manner as in case 1. The enlarged, hard and nodular brachial plexus and C8 and T1 spinal nerves were identified and followed proximally, then transected, allowing removal of the distal part of the mass. The approach to a planned C7–T2 hemilaminectomy was similar to that described in case 1, though the size of the patient made muscle dissection and retraction challenging. The C7–T2 vertebrae were identified and the hemilaminectomy was commenced but during drilling significant hemorrhage occurred from the vertebral venous sinus. The drilling of the hemilaminectomy window was incomplete at this stage and it was thought that the sinus was displaced due to the presence of the mass. Bleeding was controlled using bone wax and fibrillar collagen (Lyostypt). Given this complication, the decision was made to discontinue with the hemilaminectomy and transect the abnormal nerves as close as possible to their entry to their respective foramina. Lavage, chest drain placement and closure were performed as in case 1.

### Recovery and Follow-Up

The dog was comfortable the morning following surgery and was discharged after 4 days. At 2-week re-examination there were no neurological abnormalities present. Definitive intent radiotherapy was performed (50 Gy over 20 daily fractions). At time of writing, 7 months after surgery, the dog was coping well on three legs without any other abnormalities.

## Histopathology

All three masses showed similar histopathological features. All were moderately cellular and composed of fusiform cells arranged in intersecting bundles, streams and whorls, supported by a moderate amount of fibrovascular stroma. Neoplastic cells had indistinct borders, a small to moderate amount of pale eosinophilic cytoplasm, ovoid nuclei with finely stippled chromatin and 1–2 distinct nucleoli. There was moderate anisocytosis and anisokaryosis with 4–19 mitotic figures in 10 400 × fields. There was individual cellular and regional necrosis. There were areas of myxomatous fibrovascular stroma and peripheral infiltration of lymphocytes. In cases 1 and 3 the mass was unencapsulated whereas that in case 2 was encapsulated. In cases 1 and 2 neoplastic cells were seen extending to the borders of the nerve root samples. In case 2 neoplastic cells were also seen in adipose tissue surrounding the nerve roots. In case 3, neoplastic cells were separated from the peripheral tissue borders by a rim of adipose tissue, ~1 mm in width, with no neoplastic cells at the proximal border. The histopathological diagnosis in all three cases was malignant PNST.

## Discussion

This report demonstrates the feasibility of a lateral approach to remove PNSTs involving the C7, C8, and T1 spinal nerve and nerve roots, with intra-thoracic invasion. Crucial to the procedure is the input of a multidisciplinary team for limb amputation, thoracotomy and rib removal. Patient positioning and tilting of the table was key as it allowed optimal, adaptable positioning throughout the procedure. The lateral approach facilitated good access, particularly in the two smaller patients, allowing gross tumor resection and in case 3, complete removal.

The objective of the surgery in all cases was to achieve resection of as much of the PNST as possible, while recognizing that complete resection would be very challenging, particularly in cases with spinal cord involvement. Hence, adjunctive radiotherapy was recommended. We sought to follow the principle of oncological surgery of reducing tumor burden as far as possible, thereby increasing the efficacy of non-surgical adjuvant therapies intended to eliminate microscopic disease, and consequently reducing the likelihood of local spread or widespread metastasis (15). In case 3, the planned hemilaminectomy part of the procedure was not completed due to hemorrhage from the vertebral venous sinus, which may have been distorted or displaced. It was considered safer to control hemorrhage and resect the tumor as close as possible to the foramina, rather than persist with the hemilaminectomy. The approach to the hemilaminectomy site was challenging in this particular case. This was because of the patient's large size and muscle mass, which limited visualization and made it difficult to adequately retract the muscles. While it is possible to perform a caudal cervical or cranial thoracic hemilaminectomy in large dogs, it is considerably more difficult and time-consuming than in smaller patients.

This surgical procedure was planned to permit optimal access for efficacious tumor removal. It did not, however, lead to complete proximal resection in cases 1 and 2. It is possible that performing a durotomy would have allowed more neoplastic tissue to be removed. Durotomy in these cases, while possible, would be challenging as the extent of the hemilaminectomies had to be relatively conservative. This was due to limitations imposed by muscle mass and vascular anatomy. The vertebral venous sinuses were likely to be displaced due to the enlarged infiltrated nerve root and had to be avoided. The vertebral artery and vein located within the transverse foramen of C6 were another impediment to ventral access (16). Durotomy may have led to iatrogenic damage to the spinal cord and, given the locally invasive nature of these tumors, durotomy is unlikely to permit complete resection in cases with intramedullary invasion.

Despite the decision not to proceed with a hemilaminectomy in case 3, this was the only tumor that was completely removed. On the basis of the MRI, nerve root involvement was thought possible and the clean histological margins were unexpected. It is possible that MRI is relatively non-specific for detection of nerve root involvement. Abnormalities on electromyography (EMG) of the epaxial muscles in dogs with PNSTs have been shown to be significantly associated with nerve root or spinal cord involvement (5). Interpreting EMG findings alongside advanced imaging may, therefore, provide superior information regarding nerve root and spinal cord involvement compared to imaging alone and should ideally be performed in cases of PNST.

In order to remove the thoracic limb to permit access and visualization for the hemilaminectomy, the affected spinal nerve had to be transected, contrary to the broad principle of oncological surgery of avoiding incision into macroscopic tumor (17) which could potentially lead to tumor cell seeding. We do not have evidence of local tumor recurrence in any of our cases. Tumor seeding was therefore not documented to have occurred, despite incision into the neoplastic nerves. However, tumor regrowth may have been delayed by radiotherapy, rather than completely inhibited, and its possibility cannot be discounted, particularly in the absence of follow-up imaging.

Due to the difficulty of clean resection, radiotherapy may be valuable in cases of PNST with involvement of the vertebral canal. Radiotherapy could be considered even in cases with clean histopathological margins, due to the typical narrow margin of resection and the possibility of “skip” lesions (18). These are foci of neoplastic cells in tissue surrounding the primary mass, that are not connected to it- essentially, they are local metastases that may not be apparent on imaging or during surgery. Inadvertent damage to local tissue, including that of the central nervous system is a rare but potentially serious complication of radiation therapy in the vicinity of the spinal cord (19). Measures taken to mitigate this risk include meticulous radiotherapy planning with precisely targeted and conformed delivery, administration of multiple, low-dose fraction, and administration of anti-inflammatories to address the inflammatory component of radiation injury. These measures were taken in all our cases.

The immediate post-operative outcome in all cases was favorable as relief of pain occurred very quickly in both painful dogs (cases 1 and 2) and case 3 has continued to enjoy an excellent quality of life. In case 1 and 2, good quality of life was achieved but this was only of relatively short duration due to the development of masses in the lungs (cases 1 and 2) and abdomen (case 2). The disease-free and survival times in these two cases fell in the range that have been previously reported for surgery (4) and radiotherapy (9) alone.

It could not be determined whether the new masses in cases 1 and 2 were related to the PNSTs, as post-mortem, and histopathology were not performed. While PNSTs are typically described as of low metastatic potential, distant metastases have occasionally been reported (20), and concurrent lung masses were found in 3/24 dogs with a histologically confirmed PNST (7). It is likely that metastatic potential exists in dogs with PNST and owners should be informed of this before embarking on invasive and expensive treatment. The signs of deterioration in case 1 (i.e., vocalizing in pain) are not typical of a lung lesion so it is possible that other undetected pathology was present, or even local recurrence of the PNST, though this remains speculative in the absence of further imaging, histopathology of the lung mass, or post-mortem.

Other possible differential diagnoses for the brachial plexus lesions in these cases included neuritis and lymphoma. Neuritis was thought unlikely as this would not typically be associated with intrathoracic involvement as seen in cases 1 and 2. In case 3, the clinical signs progressed despite corticosteroid administration, making inflammatory disease less likely. Lymphoma was also a possibility, though brachial plexus lymphoma appears to be very rare in dogs. It may occur as part of a more widespread neoplastic disease (21). In our cases, PNST was the most likely differential diagnosis for a single localized mass lesion affecting the brachial plexus region. Biopsy would ideally have been performed prior to radical surgery. Biopsy was discussed with, offered to, and declined by all owners prior to proceeding with radical surgery. In cases 1 and 2, pre-referral CT, rather than MRI, was performed. It is possible that MRI might have allowed more accurate assessment of spinal cord invasion and therefore superior pre-operative planning compared to CT. On the other hand, CT does have advantages over MRI including its speed, wider availability and capacity for detailed assessment of the thorax in the same procedure. Repeat advanced imaging would ideally comprise part of the follow up in cases of PNST; this was not performed in our cases. A topic of interest with respect to advanced imaging in cases of human PNST is the use of MRI to distinguish benign from malignant lesions (22). This has not been reported in dogs, but extrapolation to canine cases merits future investigation.

At our clinic, chemotherapy has generally not previously been employed for PNSTs, due to assertions (2, 4) that these tumors are not typically widely metastatic. Local treatment has therefore been thought to be more appropriate. In case 2, chemotherapy was instituted at a different center, chosen by the owner for follow-up care for geographical reasons, and the rationale for the decision to use chemotherapy was not available to us. Unfortunately, this case developed suspected metastatic disease despite chemotherapy. It is unknown whether the use of chemotherapy had any efficacy- it may not have been beneficial; conversely, it may have delayed the onset of the suspected metastatic disease. The metastatic potential of PNST is unknown, and a large number of cases, systematically screened for metastatic disease, would be required for clear information on this.

Very little information is available regarding chemotherapy for canine PNST. Some response has been reported in isolated cases treated with nitrosylcobalamin (23), and cyclophosphamide with piroxicam (20). Chemotherapy is used in some human PNSTs, with doxorubicin and cyclophosphamide/ifosfamide showing the greatest efficacy (12, 24–26) though the evidence supporting the use of conventional chemotherapeutic agents is mixed (27). Novel molecular-targeting agents that target growth factor and other receptors involved in oncogenesis and tumor proliferation (12, 28–32) have been described, though clinical benefit has not yet been reported. Gene-based therapies, currently under investigation in a laboratory setting, are another area of interest (12). Importantly, the efficacy of chemotherapy in human PNST is profoundly affected by the patient's neurofibromatosis type 1 (NF1) tumor predisposition syndrome status (25). It has been suggested that dogs with PNST may represent a naturally occurring model of NF1, though genetic studies to confirm this are lacking (33). This presents an intriguing topic for future research. The use of chemotherapy for PNST is a subject of debate amongst human oncologists. Information on its use in canine PNST is sparse and is an area that warrants further investigation.

In conclusion, the reported cases demonstrate the feasibility of a lateral approach for resection of PNSTs involving the C7–T1 spinal nerves. The completeness of tumor resection, as well as disease-free and survival times, was variable. Two of three cases developed presumed metastasis or secondary neoplasia, which was unexpected considering previously available data on PNST. The benefit of radiotherapy in addition to surgery remains to be confirmed. A larger study is required to determine the efficacy of this treatment regime for canine PNST.

## Data Availability Statement

The raw data supporting the conclusions of this article will be made available by the authors, without undue reservation.

## Ethics Statement

Ethical review and approval was not required for the animal study because this report provides only a description of clinical care provided to patients according to best practice. Owners consented for data related to the patients to be published. Written informed consent was obtained from the owners for the participation of their animals in this study.

## Author Contributions

AU, OM, and NS were involved in the surgeries. SM planned and oversaw radiotherapy. AU, OM, NS, and SM involved in the assessment and management of the cases reported. All authors listed have made a substantial, direct, and intellectual contribution to the work and approved it for publication.

## Funding

Linnaeus Veterinary Limited supported the costs of the open access publication charges.

## Conflict of Interest

All authors were employed by company Linnaeus Veterinary Limited. The handling Editor declared a past co-authorship and past collaboration with one of the authors, OM.

## Publisher's Note

All claims expressed in this article are solely those of the authors and do not necessarily represent those of their affiliated organizations, or those of the publisher, the editors and the reviewers. Any product that may be evaluated in this article, or claim that may be made by its manufacturer, is not guaranteed or endorsed by the publisher.
